# Supplementary material for: MetaRibo-Seq measures translation in microbiomes
Source: Nat Commun. 2020 Jun 29;11:3268. doi: 10.1038/s41467-020-17081-z (PMC7324362; doi:10.1038/s41467-020-17081-z)
Supplement: Supplementary file 10 — Supplementary Data 7 [file 41467_2020_17081_MOESM10_ESM.zip › File2/Confidence_VeryHigh_Taxonomy/122182_out.krona.html]

Javascript must be enabled to view this page.

members
magnitude
magnitudeUnassigned
count
unassigned
taxon
rank

122182\_out

15

superkingdom
15
2

phylum
1224
15

1
28216
class

order
1
206351

481
1
family

1
32257
genus


SRS144237\_contig\_number\_contig-100\_3050.172441
species
1
505

class
14
1236

13
135625
order

family
13
712

genus
745
1

species
747
1

subspecies

SRS893273\_contig\_number\_contig-100\_3212.169281
44283
1

416916
12
genus

739
12
species

SRS011152\_contig\_number\_27697SRS013723\_contig\_number\_contig-100\_5065.239466SRS018157\_contig\_number\_contig-100\_2616.294680SRS022602\_contig\_number\_8817SRS022621\_contig\_number\_contig-100\_7997.7997SRS023938\_contig\_number\_5692SRS045197\_contig\_number\_11947SRS049268\_contig\_number\_contig-100\_1344.214653SRS077508\_contig\_number\_contig-100\_9102.37371SRS101388\_contig\_number\_40842SRS1055055\_contig\_number\_contig-100\_11779.98706SRS893385\_contig\_number\_contig-100\_4417.206714

order
1
135615

868
1
family

1
2717
genus

2718
1
species

SRS077861\_contig\_number\_21675
